# Genomic Instability and Carcinogenesis of Heavy Charged Particles Radiation: Clinical and Environmental Implications

**DOI:** 10.3390/medicina55090591

**Published:** 2019-09-13

**Authors:** Keywan Mortezaee, Masoud Najafi, Bagher Farhood, Amirhossein Ahmadi, Dheyauldeen Shabeeb, Ahmed Eleojo Musa

**Affiliations:** 1 Department of Anatomy, School of Medicine, Kurdistan University of Medical Sciences, Sanandaj, Iran; keywan987@yahoo.com; 2 Radiology and Nuclear Medicine Department, School of Paramedical Sciences, Kermanshah University of Medical Sciences, Kermanshah 6715847141, Iran; 3 Departments of Medical Physics and Radiology, Faculty of Paramedical Sciences, Kashan University of Medical Sciences, Kashan 8715988141, Iran; 4 Pharmaceutical Sciences Research Center, Faculty of Pharmacy, Mazandaran University of Medical Sciences, Sari 48175-861, Iran; amirhossein_pharma@yahoo.com; 5 Department of Physiology, College of Medicine, University of Misan, Misan 62010, Iraq; sazanatop5@yahoo.com; 6 Misan Radiotherapy Center, Misan Health Directorate, Ministry of Health Environment, Misan 62010, Iraq; 7 Department of Medical Physics, Tehran University of Medical Sciences (International Campus), Tehran 1416753955, Iran; ahmed.eleojo.musa@yahoo.com; 8 Department of Physics, Federal University of Technology, Minna 65, Nigeria

**Keywords:** heavy charged particles, radiation, Genomic Instability, Neoplasm, LET, Bystander Effect

## Abstract

One of the uses of ionizing radiation is in cancer treatment. The use of heavy charged particles for treatment has been introduced in recent decades because of their priority for deposition of radiation energy in the tumor, via the Bragg peak phenomenon. In addition to medical implications, exposure to heavy charged particles is a crucial issue for environmental and space radiobiology. Ionizing radiation is one of the most powerful clastogenic and carcinogenic agents. Studies have shown that although both low and high linear energy transfer (LET) radiations are carcinogenic, their risks are different. Molecular studies have also shown that although heavy charged particles mainly induce DNA damage directly, they may be more potent inducer of endogenous generation of free radicals compared to the low LET gamma or X-rays. It seems that the severity of genotoxicity for non-irradiated bystander cells is potentiated as the quality of radiation increases. However, this is not true in all situations. Evidence suggests the involvement of some mechanisms such as upregulation of pro-oxidant enzymes and change in the methylation of DNA in the development of genomic instability and carcinogenesis. This review aimed to report important issues for genotoxicity of carcinogenic effects of heavy charged particles. Furthermore, we tried to explain some mechanisms that may be involved in cancer development following exposure to heavy charged particles.

## 1. Introduction

For many years, the use of heavy charged particles has been proposed for clinical cancer therapy because of their biophysical properties. For the first time, in 1940, the neutron was the first particle used for cancer radiotherapy [1]. Several years after, the use of protons and heavier particles were incorporated into some oncology centers. Nowadays, there is a growing number of centers using particle radiotherapy. It is estimated that more than one hundred thousand patients are treated with protons or other particle types [2]. The main factor behind the use of heavy charged particles is their interesting biophysical properties which cause lower radiation dose deposition in surrounding normal tissues while delivering most of its energy to the tumor [3]. However, their high cost in comparison to X-ray technology is a barrier for establishing new centers utilizing charged particles [4].

In addition to clinical importance, knowledge of the radiobiological properties of charged particles is important for understanding the environmental impacts of ionizing radiation. Radon, the main source of alpha particles, has been proposed as a contributing factor to the incidence of lung cancer due to its high background radiation. The carcinogenic effect of alpha particles derived from radon gas has been confirmed many years ago among miners [5]. Nowadays, inhalation of radon gas is the second reason for incidence of lung cancer [6,7,8].

Another important source of heavy charged particles is space, which itself is composed of galactic cosmic and solar radiation particles. The particles which originate from galactic cosmic rays are mainly heavy particles such as iron, which have high energy up to 10^17^ eV [9]. However, the energy of solar particles which are mainly proton particles may be in the range of 80 MeV up to more than 1 GeV [10]. Space projects by some countries in recent decades give new insights in radiobiology, a concept known as space radiobiology. The major challenge in space radiobiology is the prolonged exposure to high energy heavy particles which may affect astronaut’s health and safety. This is a challenging topic in radiobiology; hence, several projects have been funded to investigate the biological effects of exposure to high energy particles during deep space or Mars exploration [11].

## 2. Interactions of Heavy Charged Particles with Cells

The most interesting property of heavy radiation particles is the change in the linear energy transfer (LET) when penetrating tissues. Particles deposit lower energies in the first layers, while they can deposit their remaining energies at special depths. These depths depend on particle energy, mass, and charge. The higher masses of charged particles compared to electron causes low energy deposition along their track. However, higher charge and lower energy lead to more energy deposition. These properties of heavy charged particles cause lower energy deposition in the first range of particles, while they deposit most of their energies at the end of their range. The end of a particle’s path that receives high energy is known as the Bragg peak [4]. After Bragg peak, low energy may be deposited. However, for heavier particles such as carbon, silicon, and iron ions, higher doses of radiation can be deposited after the Bragg peak. This results in splitting up of particles, leading to the formation of some lower weight fragments. In this situation, fragmentation is responsible for dose deposition behind the peak [12].

## 3. The Clinical Importance of Heavy Charged Particles

Due to the higher efficiency of heavy charged particles compared to X-rays, there is an increasing interest in the treatment of cancers using charged particles. Nowadays, low LET X-rays are the most common radiation modality for the treatment of cancer in clinical oncology. Interaction of low LET gamma or X-rays with cancer cells cause several interactions with genomic contents of cancer cells; however, the main mechanism of cell death by low LET radiations is free radical production following radiolysis of water molecules [13]. Low LET radiations deposit a large amount of their energy in surrounding normal tissues. Therefore, there is a need for different radiation fields for sparing normal tissues [4]. One of the main concerns for tumor irradiation with low LET radiations is the high probability of tumor recurrence. This is as a result of the lower ratio of lethal damages in cancer cells following therapy with X-rays [2]. By contrast, irradiation with high LET radiations causes massive DNA damage and lethal effects, leading to reduced possibility of DNA repair [14]. The inhibition of DNA damage responses (DDRs) can improve the efficiency of heavy charged particles in suppressing tumor regression [15].

One of the most important properties of heavy charged particles compared to low LET X-rays is the lower dependency on the oxygen level of cells. This is an interesting issue because some clonogenic cancer cells are located within the hypoxic area of tumors. For low LET X-rays, the therapeutic efficiency is highly dependent on the presence of oxygen molecules. However, for high LET particles, oxygen has a lower effect on cell survival. Heavy charged particles have more ability to kill hypoxic cancer cells via direct interaction with DNA without the need for a free radical generation [16]. Using charged particle radiations for tumor therapy can reduce the volume of irradiated normal tissues, leading to more protection. This is because of the complete energy falloff in tumor volume and complete protection of organs behind. Furthermore, if the radiation field is planned correctly, irradiation with heavy charged particles do not cause damage to the adjacent tissues. This issue is an important concern for radiotherapy with low LET X-rays when the tumor is located within or next to a radiosensitive organ. For example, toxicity of spermatogenesis following prostate cancer radiotherapy, or thyroid damage after radiotherapy for breast cancer are major side effects for patients with these cancers [17,18]. In addition to acute reactions in normal tissues, lower incidence of second primary cancer some years after radiotherapy is another possible advantage of radiotherapy with charged particle radiations [19].

Although some advantages of using radiation particles compared to X-rays have been confirmed for several types of cancers, the cost-effectiveness of this modality is a challenge for the development of new treatment centers with protons or other charged particle radiations [20]. Some studies have proposed that heavy charged particles have a higher relative biological effectiveness (RBE) compared to X-rays [21]. Also, when a radiosensitive organ is within the radiation field, it may show more acute reactions for charged particle radiation compared to X-rays [22].

## 4. The Environmental Importance of Heavy Charged Particles

Environmental radiation is responsible for most radiation exposures to people worldwide. However, in the past two decades, the main source of radiation exposure for some developed countries such as the United States and Japan was from medicine [23]. The main source of environmental radiation is natural background radiations which originate from uranium and thorium fissures. Radon-222 is one of the products from uranium and thorium and is responsible for most radiation doses received by people from the environment [23]. Radon enters the lung during breathing, in which several radon atoms decay to alpha particles and also produce other radioactive particles such as polonium and lead (Pb-210). These particles can be absorbed by or irradiate other organs like bone marrow and gastrointestinal system. Pb-210 has a long half-life and can inter the red blood cells and bones, thereby irradiating these cells for a long time [24,25].

As earlier mentioned, the interaction of heavy charged particles such as iron, carbon, and silicon with different materials lead to the formation of lower weight fragments. These fragments have lower masses with different charges. The lower mass of particles causes more penetration of fragments compared to heavier particles. This issue is very important for shielding against heavy charged particles, such as in space crafts. Galaxy and solar origin particles have very high energy that are able to penetrate into space crafts, which lead to exposure of astronauts to various types of ionizing radiation. Genotoxicity of charged particle radiation is an important issue for space missions. The most frequent particle beyond the earth’s atmosphere is proton, which originates from solar winds. However, other heavier particles such as helium, oxygen and iron ions that originate from the galaxy are responsible for genotoxicity in spacecraft passengers [13]. Some studies have suggested that DNA rearrangement following exposure to heavy charged particles could play a role in adaptive response and evolution [26]. This issue may be important for space mission. However, it has been suggested that cosmic radiation may be involved in the evolution of the earth [27].

## 5. Genomic Instability of Heavy Charged Particles

Genomic instability is the permanent change in the genomic contents that lead to heritable mutations. Evidence from studies indicates that exposure to ionizing radiation cause mutation in progeny cells. In vivo studies have also shown that exposure to radiation can trigger carcinogenesis in subsequent generations. To date, numerous studies have shown that charged particle radiation is able to induce chromosomal aberrations and genomic instability in irradiated cells as well as their progenies [28,29,30]. Induction of mutation by heavy charged particles has been revealed by several studies [31,32]. It has also been confirmed that heavy charged particles are more potent genotoxic agents compared to gamma or X-rays [33]. It seems that mutations and genotoxicity depend on both LET and type of irradiated tissue [33]. A study by Masumura et al. evaluated the frequency of mutation in different tissues of mice following irradiation with carbon, X-rays and gamma rays. They showed that the frequency of mutations including point mutation and deletion are higher after carbon ion exposure in liver, spleen, and kidney compared to low LET gamma or X-rays. Interestingly, carbon particles did not cause any significant deletion or point mutation in the testis. Furthermore, gpt mutation was less for carbon particles. Results indicated that heavy carbon particles mainly induce double-strand breaks, while low LET radiations cause oxidative DNA injury [33].

Although the genotoxic effect of charged particle radiation is mainly mediated via direct interaction with DNA, it seems that endogenous production of ROS after DNA damage and cell death plays a key role in genotoxicity by high LET radiations. This has been confirmed in experimental studies. Exposure to iron-56 particles has been shown to induce more DNA injury compared to gamma rays [22]. On the other hand, long-time evaluations showed that irradiation of mice intestine and colon with carbon particles cause more production of ROS compared to gamma rays [34]. Similar effects have been shown for some days to weeks after exposure [35,36,37]. It seems that charged particle radiation is able to change the metabolism in irradiated organs more effectively compared to low LET radiation, leading to more chronic oxidative stress [38]. Increasing metabolism has also been observed following exposure to high energy protons [39]. Comparison of the genotoxic effects of iron and proton particles in mice kidney has shown that iron particles are very genotoxic compared to proton particles [34].

The main sources of ROS generation following exposure to ionizing radiation include mitochondria, NADPH oxidase family, cyclooxygenase-2 (COX-2), inducible nitric oxide synthase (iNOS), and lipoxygenases (LOX). However, the expression of each of these enzymes and also an overproduction of superoxide by mitochondria is a tissue-specific phenomenon and need further investigations by experimental studies. The overexpression of these pro-oxidants is mediated following unrepaired DNA damage and cell death. In this situation, massive necrosis and apoptosis trigger activation of macrophages and lymphocytes in response to released danger alarms such as high mobility group box 1 (HMGB1) [40]. Danger alarms trigger macrophages and Lymphocyte-T to induce transcription factors such as STAT-3 and nuclear factor-kappa B (NF-kB) [41]. The role of MAPK p38 in chronic oxidative stress following exposure to highly charged particles has also been proven [42]. These transcription factors regulate the expression of pro-oxidant enzymes and also a wide range of cytokines and growth factors [43]. The increased level of some cytokines and growth factors such as IL-1, IL-8, TGF-*β* and insulin-like growth factor 1 (IGF1) can further amplify redox activation, which may lead to chronic oxidative stress [44,45]. In an experimental study, the role of high energy iron particles on the activation of redox response in mice intestine was examined. Mice intestine was irradiated with high energy iron particles or gamma rays. Intracellular free radicals were measured after 1 year. Results showed a remarkable increase in ROS and superoxide generation for mice exposed to iron particles. However, only a mild increase in ROS, but not superoxide generation was confirmed for mice exposed to gamma rays. In addition, an increased level of nitric oxide (NO) generation and reduced mitochondrial membrane potential, as well as upregulation of NADPH oxidase, were more obvious for mice irradiated with iron particles [46]. Similar results were observed for carbon and some other heavy charged particles [34,47,48].

One of the important mechanisms of genomic instability and increased risk of carcinogenesis is a change in epigenetic modulators that affect DNA methylation. Hypermethylation of tumor suppressor genes leads to inhibition of their activities while increasing the activities of oncogenes. Furthermore, hypomethylation may stimulate the upregulation of oncogenes [49,50]. The effects of low LET X-rays on methylation of DNA has been reported by several in vitro and in vivo studies [51,52]. However, the effect of heavy charged particles on epigenetic changes including DNA methylation is very limited. A study by Kennedy et al. evaluated the effect of high LET iron and silicon ions, and also low LET X-rays on global methylation of human bronchial epithelial cells. The detection of CpG sites showed that all types of radiation can cause both hyper- and hypomethylation in CpG sites. For iron ions, nearly 90% of changes were due to hypermethylation. Furthermore, the methylation was increased as the dose of iron particles increased. However, for silicon ions the levels of both hyper- and hypomethylation were close; hence, no trend was observed following an increase in radiation dose. Similar to previous studies, exposure to low LET X-rays led to global hypomethylation, which has a direct relation with radiation dose [53].

It has been shown that irradiation with heavy charged particles can induce changes in the expression of some genes that may trigger abnormal proliferation and tumorigenesis. As earlier mentioned, irradiation of mice intestine led to the persistent generation of free radicals [46]. It has also been shown that irradiating mice intestine with iron particles caused persistent upregulation of Wnt/*β*-catenin signaling pathway, which plays a key role in proliferation and migration of epithelial cells in the intestine. Exposure to iron particles increased proliferation, while migration in intestinal epithelial cells was reduced. These changes were associated with a remarkable increase in DNA damage and senescence [54]. As senescence is the main stimulator of NADPH oxidase gene upregulation, it is possible that exposure to heavy charged particles induces chronic upregulation of NADPH oxidase and continuous generation of ROS via induction of senescence [55].

## 6. Evidence on Carcinogenic Effects of Heavy Charged Particles

In addition to DNA damage and genomic instability, experimental studies have confirmed the direct effect of charged particles on tumor induction. In an in vivo study, Ando et al. evaluated tumor induction following local mice leg irradiation with carbon ions or gamma rays. Results of this study showed that although carbon ions are able to induce a tumor, the risk of carcinogenesis from carbon ions is not seriously higher than gamma rays [56]. By contrast, another study suggested that exposure to carbon ions has a higher risk for induction and also metastasis of mammary carcinoma in rats [57]. Another interesting study by Ando et al. tried to explain the role of RBE in tumor induction by carbon ion particles. They used gamma rays and carbon ions of different LETs, including 15, 45 and 75 keV/μm. Similarly, in another study they irradiated mice legs and detected tumor induction for all life spans. Interestingly, their results showed that tumor induction has a direct relation with LET of carbon ions. They proposed that carbon ions with 15 keV/μm have lower RBE for tumor induction compared to gamma rays, while carbon ions with higher LET are associated with higher risk. RBE for tumor induction with carbon ions with 75 keV/μm was greater than 2 [58]. Similar results were observed when mice intestine was irradiated with different types of ions including silicon, carbon, and iron [28,59].

### 6.1. Bystander Effects

Ta Bystander effect is an interesting phenomenon in modern radiobiology. It is an indirect effect of ionizing radiation which causes damages to non-irradiated cells. Bystander effect results from the release of clastogenic factors from damaged cells, which can change gene expression and trigger overproduction of endogenous free radicals [60]. For the first time, Nagasawa showed that irradiation with protons causes sister chromatid exchanges in Chinese hamster ovary (CHO) cells [61]. Studies further confirmed the clastogenic effect of proton or other particle radiation types on bystander cells [62]. It has been suggested that intercellular communications such as gap junctions play a key role in the induction of DNA damage and oxidative stress in bystander cells [63,64].

It seems that migration of danger alarms from injured or dead cells to bystander cells triggers inflammation and endogenous ROS and NO production following upregulation of some mediators such as MAPKs, protein kinase C (PKC), DNA-PKCs, NF-kB, cyclooxygenase-2 (COX-2), inducible nitric oxide synthase (iNOS), and NADPH oxidase [65,66,67,68,69,70,71]. These changes may be observable following radiation interaction with the membrane, a non-critical target of cells [72]. Some studies have been conducted to evaluate the role of LET in radiation-induced injury in bystander cells. In some studies, it has been shown that high LET particles induce more damage to bystander cells compared to protons or X-rays showed [73,74]. Moreover, for prostate cancer cells, alpha particles have shown lower toxicity compared to X-rays [75].

A study by Autsavapromporn et al. attempted to detect micronuclei formation in bystander human fibroblast cells following irradiation with different LET radiations. They irradiated cells with carbon, neon, argon or X-rays. Also, they used 18-*α*-glycyrrhetinic acid, which inhibits gap junctions, for evaluating the role of gap junctions in bystander effect by high LET particles. Results showed that high LET particles are able to induce more micronuclei formation compared to low LET. Interestingly, bystander induction by high LET has a stronger relation with gap junctions. Inhibition of gap junctions reduced micronuclei formation in a LET dependent manner, while no significant reduction was observed for X-rays [76]. By contrast, a study by Shao et al. proposed that micronuclei induction in human fibroblast cells following local irradiation with neon or argon ions is the same. They also showed that micronuclei formation has a direct relation with the number of irradiated cells and the number of particles. Inhibition of gap junctions alone did not show a remarkable reduction in micronuclei formation, while for cells incubated with ROS scavenger and an inhibitor of gap junction, micronuclei formation decreased significantly [77]. It was observed that using a free radical scavenger can suppress upregulation of some pro-oxidant enzymes like COX-2 in bystander cells, which plays a key role in genotoxicity in bystander cells [78]. It was suggested that COX-2 upregulation has a direct relation with radiation quality [78].

A key mechanism for prolonged oxidative stress is permanent changes in genomic contents, which cause damages to the progeny of irradiated and bystander cells. It seems that high LET particles via this mechanism are able to induce longer-term oxidative stress and genotoxicity in both irradiated and non-irradiated cells [65,79]. Irradiation with different LET particles including proton with 0.2 keV/μm to silicon and iron particles with 51 and 151 keV/μm showed that low LET protons do not cause persistent endogenous ROS production, while higher LET particles via changes in the metabolic and redox responses induce chronic oxidative stress in the next generation of bystander cells [80]. Similarly, neoplastic transformation has been observed in the progeny of bystander cells after irradiation with high LET particles, but not for lower LET protons [81]. Induction of oxidative stress in the progeny of bystander cells is positively influenced by the number of gap junctions [82]. Results of molecular studies showed that stimulation of mir-21 and TGF-*β*1-Smad2 is involved in oxidative stress and DNA damage in bystander cells following irradiation with protons. However, these changes have not been shown for X-rays [74].

Another key player in free radical production following exposure to radiation is mitochondrial malfunction and changes in cellular metabolism. Numerous experimental studies have shown that the mitochondria play a key role in chronic oxidative stress and genomic instability in both irradiated and bystander cells. It seems that radiation interaction and oxidative stress lead to mutations in mitochondrial DNA (mtDNA). During normal conditions, approximately 5% of mitochondria yield of oxidative phosphorylation is superoxide that is neutralized by mitochondrial superoxide dismutase (mtSOD). However, following mutation in mtDNA, the yield of superoxide from mitochondria is increased, which lead to overwhelming antioxidant defense. In this situation, chronic oxidative stress is probable because of mitochondrial malfunction, which is also associated with increased activity of other pro-oxidant enzymes such as iNOS, COX-2 and NADPH oxidase. RNA-sequencing analysis has also shown that irradiating the cytoplasm with alpha ions would lead to changes in mitochondrial metabolism. Results revealed a remarkable increase in the expression of genes involved in glycolytic pathways, including PGC-1*α* and Pim-1 kinase. These changes were sustained even 2 weeks after irradiation. As Pim-1 kinase inhibits AMPK, a key player in DNA repair and cell response to stress conditions, these changes may promote genomic instability and transformation in normal irradiated and bystander cells [83].

### 6.2. Non-Targeted (Distant Bystander) Effect

**Figure 1:**
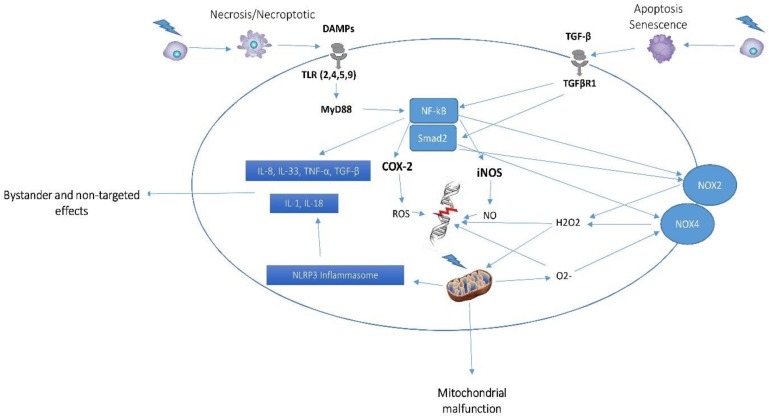
Mechanisms of genomic instability and carcinogenesis of heavy charged particles. Exposure to heavy charged particles induces different types of cell death. Apoptosis and senescence trigger activation of TGF-*β* and its downstream pro-oxidant enzymes such as NOX2 and NOX4. However, necrosis and necroptosis induce inflammatory responses via upregulation of NF-kB, COX-2, and iNOS. Generated ROS by NADPH oxidase enzymes and COX-2 can attack DNA, leading to DNA damage. Furthermore, NO production by iNOS can damage to DNA and also suppress repair mechanisms. Exposure of mitochondria to radiation or generated ROS by pro-oxidant enzymes cause mitochondria malfunction and continuous production of superoxide. Increased production of inflammatory cytokines may lead to chronic oxidative stress and genomic instability in bystander cells/tissues. TGF-*β:* Transforming growth factor beta; NOX: NADPH Oxidase; NF-kB: Nuclear Factor kappa B; COX-2: Cyclooxygenase-2; iNOS: Inducible nitric oxide synthase; DAMPs: Damage-associated molecular patterns; TLR: Toll-like receptor; MyD88: Myeloid differentiation primary response 88.

Non-targeted effect of radiation is similar to the bystander effect, but it occurs in distant organs following local irradiation of a body part. This is an important effect that can increase the risk of carcinogenesis in radiotherapy patients as well as persons exposed to accidental radiation events. Evidence from patients who underwent radiotherapy and also Chernobyl survivors reveal the presence of some clastogenic factors in their serum. Co-treatment of healthy cells with serum of these people cause DNA aberrations in non-irradiated cells, which indicate existence of some released factors from irradiated cells that are able to attack genomic contents of non-irradiated cells in distant organs. Although studies investigating the role of non-targeted effect in genomic instability following exposure to charged radiation particles are few, there is evidence for pivotal roles of radiation-induced inflammatory responses. Irradiating the lower part of rat’s lung has been shown to cause significant micronuclei formation that is associated with increased macrophage activity and release of pro-inflammatory cytokines such as IL-1, IL-6, TNF-*α* and TGF-*β* [84,85]. Irradiation of the lower abdominal parts in gpt delta mice has been shown to cause more than 3-fold increase in the expression of COX-2 in breast tissue, which was associated with a remarkable augment in ROS production and double-strand break. Direct irradiation also led to similar effects [86] (Table 1, Figure 1).

## 7. Conclusions

For many years, it has been confirmed that increased risk of carcinogenesis is one of the most critical side effects of ionizing radiation. Studies have been conducted to investigate the risks as well as benefits of using heavy charged particles for cancer therapy compared to low LET radiation. Results of experimental studies have shown that although heavy charged particles have higher risks compared to low LET radiation for inducing cancers, it seems that for some other types of cancers this may not be true. Other important implications of heavy charged particles are environmental and space radiation toxicities. Radon and its daughters are the main sources of environmental radiation and are responsible for some lung cancers. Molecular studies suggest that although some mechanisms of heavy charged particles are similar to low LET radiation, heavy charged particles are able to induce endogenous production of free radicals more potently. Furthermore, heavy charged particles may have different effects on epigenetic modulation such as methylation of DNA following irradiation. In addition to direct effects on irradiated cells, heavy charged particles have been shown to be genotoxic to non-irradiated bystander cells. While it is possible that proton has lower toxicity compared to gamma or X-rays for bystander cells. It is suggested that mitochondrial malfunction, which leads to superoxide overproduction is the main reason for genotoxicities in both irradiated and bystander cells.

## Figures and Tables

**Table 1:** Summary results of genotoxicity effect of radiation particles.

Route	Tissues/Cells	Radiation Type	Dose	Findings	Reference
Mice	Liver, spleen, kidney, testis	Carbon	10 Gy	Carbon radiation caused higher Spi mutations and lower gpt mutations. compared to X-rays. Mutations by carbon radiation were tissue-specific.	[33]
Mice	Intestine	Iron particles	1.6 Gy	Irradiation with iron particle led to the chronic generation of nitric oxide (probably by iNOS), superoxide and ROS generation by mitochondria and NADPH oxidase.	[46]
In vitro	Human bronchial epithelial cells	Iron and silicon ions	0–1 Gy	Unlike X-rays, iron charged particles caused remarkable. hypermethylation, while exposure to silicon ions led to both hyper- and hypomethylation.	[53]
Mice	Leg	Carbon ions	65 Gy	Carbon ions had no higher risk ions for second cancers in legs compared to gamma rays.	[56]
Rat	Whole-body	Carbon ions	0.5–2 Gy	Rats showed a higher risk of mammary carcinoma following exposure to carbon ions compared to gamma rays.	[57]
Mice	Leg	Carbon ions	50 Gy	Carbon ions with lower LET may be safer for tumor induction, while higher LET has a higher risk for tumorigenesis.	[58]
Mice	Intestine	Iron ions	1.6 Gy	Iron particle caused more tumor induction in the intestine compared to gamma rays.	[28]
In vitro	Human fibroblast cells	Carbon, neon, argon or X-rays	0–1 Gy	The micronuclei formation in bystander cells was dependent on LET. Higher LET has a stronger relation with gap junctions.	[76]
In vitro	Human fibroblast cells	Neon or argon	0–4 particles	Micronuclei formation has a direct relation with the number of particles. Inhibition of gap junctions alone did not reduce micronuclei formation.	[77]
In vitro	AG1522	Proton, silicon, and iron	0–2 Gy	Higher LET particles are able to induce redox reactions more effectively.	[80]
In vitro	Keratinocytes	Alpha particles	0–10 Gy	Upregulation of mir-21 and TGF-β1-Smad2 pathways are involved in chronic oxidative stress in bystander cells.	[74]
Gpt delta mice	Breast tissue	Argon or carbon	4.5 Gy for carbon and 1.5 Gy for argon	Local abdominal irradiation caused ROS production via stimulation of COX-2.	[86]

## References

[1] Stone R.S. (1948). Neutron therapy and specific ionization. Am. J. Roentgenol. Radium..

[2] Mitin T., Zietman A.L. (2014). Promise and Pitfalls of Heavy-Particle Therapy. J. Clin. Oncol..

[3] Desouky O., Zhou G. (2016). Biophysical and radiobiological aspects of heavy charged particles. J. Taibah Univ. Sci..

[4] Skarsgard L.D. (1998). Radiobiology with heavy charged particles: A historical review. Phys. Med..

[5] Lantz P.M., Mendez D., Philbert M.A. (2013). Radon, smoking, and lung cancer: The need to refocus radon control policy. Am. J. Public Health.

[6] Kim J.-H., Ha M. (2018). The Disease Burden of Lung Cancer Attributable to Residential Radon Exposure in Korean Homes. J. Korean Med. Sci..

[7] Peterson E., Aker A., Kim J., Li Y., Brand K., Copes R. (2013). Lung cancer risk from radon in Ontario, Canada: How many lung cancers can we prevent?. Cancer Causes Control.

[8] Sethi T.K., El-Ghamry M.N., Kloecker G.H. (2012). Radon and lung cancer. Clin. Adv. Hematol. Oncol..

[9] Thoudam S., Rachen J., van Vliet A., Achterberg A., Buitink S., Falcke H., Hörandel J. (2016). Cosmic-ray energy spectrum and composition up to the ankle: The case for a second Galactic component. Astron. Astrophy..

[10] Adriani O., Barbarino G., Bazilevskaya G., Bellotti R., Boezio M., Bogomolov E., Bongi M., Bonvicini V., Bottai S., Bravar U. (2015). Pamela’s measurements of magnetospheric effects on high-energy solar particles. Astrophys. J. Lett..

[11] Simon M., Latorella K., Martin J., Cerro J., Lepsch R., Jefferies S., Goodliff K., Smitherman D., McCleskey C., Stromgren C. NASA’s Advanced Exploration Systems Mars Transit Habitat Refinement Point of Departure Design. Proceedings of the 2017 IEEE Aerospace Conference.

[12] Allen C., Borak T.B., Tsujii H., Nickoloff J.A. (2011). Heavy charged particle radiobiology: Using enhanced biological effectiveness and improved beam focusing to advance cancer therapy. Mutat. Res..

[13] Blakely E.A., Kronenberg A. (1998). Heavy-ion radiobiology: New approaches to delineate mechanisms underlying enhanced biological effectiveness. Radiat. Res..

[14] Zhu J., Ren Z., Chen Y., Hu B. (2016). The biological effects induced by high-charged and energy particles and its application in cancer therapy. Int. J. Radiat. Res..

[15] Suman S., Moon B.H., Thakor H., Fornace A.J., Datta K. (2014). Wip1 abrogation decreases intestinal tumor frequency in APC(Min/+) mice irrespective of radiation quality. Radiat. Res..

[16] Tinganelli W., Durante M., Hirayama R., Krämer M., Maier A., Kraft-Weyrather W., Furusawa Y., Friedrich T., Scifoni E. (2015). Kill-painting of hypoxic tumours in charged particle therapy. Sci. Rep..

[17] Farhood B., Mortezaee K., Haghi-Aminjan H., Khanlarkhani N., Salehi E., Nashtaei M.S., Najafi M., Sahebkar A. (2019). A systematic review of radiation-induced testicular toxicities following radiotherapy for prostate cancer. J. Cell. Physiol..

[18] Mortezaee K., Ahmadi A., Haghi-Aminjan H., Khanlarkhani N., Salehi E., Shabani Nashtaei M., Farhood B., Najafi M., Sahebkar A. (2019). Thyroid function following breast cancer chemotherapy: A systematic review. J. Cell. Biochem..

[19] Pompos A., Durante M., Choy H. (2016). Heavy Ions in Cancer Therapy. JAMA Oncol..

[20] Held K.D., Kawamura H., Kaminuma T., Paz A.E.S., Yoshida Y., Liu Q., Willers H., Takahashi A. (2016). Effects of Charged Particles on Human Tumor Cells. Front. Oncol..

[21] Combs S.E., Debus J. (2013). Treatment with heavy charged particles: Systematic review of clinical data and current clinical (comparative) trials. Acta Oncol..

[22] Brooks A., Bao S., Rithidech K., Couch L.A., Braby L.A. (2001). Relative effectiveness of HZE iron-56 particles for the induction of cytogenetic damage in vivo. Radiat. Res..

[23] Valentin J. (2007). The 2007 Recommendations of the International Commission on Radiological Protection. ICRP publication 103. Ann. ICRP.

[24] Scott B.R. (2011). Residential radon appears to prevent lung cancer. Dose-Response A Public. Int. Hormesis Soc..

[25] Seiler R.L., Wiemels J.L. (2012). Occurrence of 210Po and biological effects of low-level exposure: The need for research. Environ. Health Perspect..

[26] Yang C.H., Craise L.M., Durante M., Mei M. (1994). Heavy-ion induced genetic changes and evolution processes. Adv. Space Res..

[27] Todd P. (1994). Cosmic radiation and evolution of life on earth: Roles of environment, adaptation and selection. Adv. Space Res..

[28] Datta K., Suman S., Kallakury B.V., Fornace A.J. (2013). Heavy ion radiation exposure triggered higher intestinal tumor frequency and greater beta-catenin activation than gamma radiation in APC(Min/+) mice. PLoS ONE.

[29] Hada M., Georgakilas A.G. (2008). Formation of clustered DNA damage after high-LET irradiation: A review. J. Radiat. Res..

[30] Hagiwara Y., Oike T., Niimi A., Yamauchi M., Sato H., Limsirichaikul S., Held K.D., Nakano T., Shibata A. (2019). Clustered DNA double-strand break formation and the repair pathway following heavy-ion irradiation. J. Radiat. Res..

[31] Yatagai F., Nohmi T., Kusakabe M., Masumura K., Yoshiki A., Yamaguchi H., Kurobe T., Kuniya K., Hanaoka F., Yano Y. (2000). Mutation induction by heavy ion irradiation of gpt delta transgenic mice. Biol. Sci. Space.

[32] Yatagai F., Kurobe T., Nohmi T., Masumura K., Tsukada T., Yamaguchi H., Kasai-Eguchi K., Fukunishi N. (2002). Heavy-ion-induced mutations in the gpt delta transgenic mouse: Effect of p53 gene knockout. Environ. Mol. Mutagen..

[33] Masumura K., Kuniya K., Kurobe T., Fukuoka M., Yatagai F., Nohmi T. (2002). Heavy-ion-induced mutations in the gpt delta transgenic mouse: Comparison of mutation spectra induced by heavy-ion, X-ray, and gamma-ray radiation. Environ. Mol. Mutagen..

[34] Suman S., Kumar S., Fornace A.J., Datta K. (2018). The effect of carbon irradiation is associated with greater oxidative stress in mouse intestine and colon relative to gamma-rays. Free Radic. Res..

[35] Dettmering T., Zahnreich S., Colindres-Rojas M., Durante M., Taucher-Scholz G., Fournier C. (2015). Increased effectiveness of carbon ions in the production of reactive oxygen species in normal human fibroblasts. J. Radiat. Res..

[36] Suman S., Rodriguez O.C., Winters T.A., Fornace A.J., Albanese C., Datta K. (2013). Therapeutic and space radiation exposure of mouse brain causes impaired DNA repair response and premature senescence by chronic oxidant production. Aging (Albany NY).

[37] Kumar S., Suman S., Fornace A.J., Datta K. (2018). Space radiation triggers persistent stress response, increases senescent signaling, and decreases cell migration in mouse intestine. Proc. Natl. Acad. Sci. USA.

[38] Ghosh S.P., Singh R., Chakraborty K., Kulkarni S., Uppal A., Luo Y., Kaur P., Pathak R., Kumar K.S., Hauer-Jensen M. (2013). Metabolomic changes in gastrointestinal tissues after whole body radiation in a murine model. Mol. Biosyst..

[39] Laiakis E.C., Trani D., Moon B.H., Strawn S.J., Fornace A.J. (2015). Metabolomic profiling of urine samples from mice exposed to protons reveals radiation quality and dose specific differences. Radiat. Res..

[40] Yoshimoto Y., Oike T., Okonogi N., Suzuki Y., Ando K., Sato H., Noda S.E., Isono M., Mimura K., Kono K. (2015). Carbon-ion beams induce production of an immune mediator protein, high mobility group box 1, at levels comparable with X-ray irradiation. J. Radiat. Res..

[41] Genard G., Wera A.-C., Huart C., Le Calve B., Penninckx S., Fattaccioli A., Tabarrant T., Demazy C., Ninane N., Heuskin A.-C. (2018). Proton irradiation orchestrates macrophage reprogramming through NFκB signaling. Cell Death Dis..

[42] Werner E., Wang H., Doetsch P.W. (2014). Opposite roles for p38MAPK-driven responses and reactive oxygen species in the persistence and resolution of radiation-induced genomic instability. PLoS ONE.

[43] Hellweg C.E., Spitta L.F., Koch K., Chishti A.A., Henschenmacher B., Diegeler S., Konda B., Feles S., Schmitz C., Berger T. (2018). The Role of the Nuclear Factor κB Pathway in the Cellular Response to Low and High Linear Energy Transfer Radiation. Int. J. Mol. Sci..

[44] Sridharan D.M., Asaithamby A., Bailey S.M., Costes S.V., Doetsch P.W., Dynan W.S., Kronenberg A., Rithidech K.N., Saha J., Snijders A.M. (2015). Understanding cancer development processes after HZE-particle exposure: Roles of, R.O.S.; DNA damage repair and inflammation. Radiat. Res..

[45] Alwood J.S., Tran L.H., Schreurs A.S., Shirazi-Fard Y., Kumar A., Hilton D., Tahimic C.G.T., Globus R.K. (2017). Dose- and Ion-Dependent Effects in the Oxidative Stress Response to Space-Like Radiation Exposure in the Skeletal System. Int. J. Mol. Sci..

[46] Datta K., Suman S., Kallakury B.V.S., Fornace A.J. (2012). Exposure to heavy ion radiation induces persistent oxidative stress in mouse intestine. PLoS ONE.

[47] Baulch J.E., Craver B.M., Tran K.K., Yu L., Chmielewski N., Allen B.D., Limoli C.L. (2015). Persistent oxidative stress in human neural stem cells exposed to low fluences of charged particles. Redox Biol..

[48] Tseng B.P., Giedzinski E., Izadi A., Suarez T., Lan M.L., Tran K.K., Acharya M.M., Nelson G.A., Raber J., Parihar V.K. (2014). Functional consequences of radiation-induced oxidative stress in cultured neural stem cells and the brain exposed to charged particle irradiation. Antioxid. Redox Signal..

[49] Luczak M.W., Jagodzinski P.P. (2006). The role of DNA methylation in cancer development. Folia Histochem. Cytobiol..

[50] Kulis M., Esteller M. (2010). DNA methylation and cancer. Adv. Genet..

[51] Mothersill C., Seymour C. (2012). Are Epigenetic Mechanisms Involved in Radiation-Induced Bystander Effects?. Front. Genet..

[52] Kovalchuk O., Baulch J.E. (2008). Epigenetic changes and nontargeted radiation effects—Is there a link?. Environ. Mol. Mutagen..

[53] Kennedy E.M., Powell D.R., Li Z., Bell J.S.K., Barwick B.G., Feng H., McCrary M.R., Dwivedi B., Kowalski J., Dynan W.S. (2018). Galactic Cosmic Radiation Induces Persistent Epigenome Alterations Relevant to Human Lung Cancer. Sci. Rep..

[54] Chai Y., Lam R.K., Calaf G.M., Zhou H., Amundson S., Hei T.K. (2013). Radiation-induced non-targeted response in vivo: Role of the TGFbeta-TGFBR1-COX-2 signalling pathway. Br. J. Cancer.

[55] Sakai Y., Yamamori T., Yoshikawa Y., Bo T., Suzuki M., Yamamoto K., Ago T., Inanami, O. (2018). NADPH oxidase 4 mediates ROS production in radiation-induced senescent cells and promotes migration of inflammatory cells. Free Radic. Res..

[56] Ando K., Koike S., Oohira C., Ogiu T., Yatagai F. (2005). Tumor induction in mice locally irradiated with carbon ions: A retrospective analysis. J. Radiat. Res..

[57] Imaoka T., Nishimura M., Kakinuma S., Hatano Y., Ohmachi Y., Yoshinaga S., Kawano A., Maekawa A., Shimada Y. (2007). High relative biologic effectiveness of carbon ion radiation on induction of rat mammary carcinoma and its lack of H-ras and Tp53 mutations. Int. J. Radiat. Oncol. Biol. Phys..

[58] Ando K., Koike S., Ohmachi Y., Ando Y., Kobashi G. (2014). Tumor induction in mice after local irradiation with single doses of either carbon-ion beams or gamma rays. Int. J. Radiat. Biol..

[59] Suman S., Kumar S., Moon B.H., Strawn S.J., Thakor H., Fan Z., Shay J.W., Fornace A.J., Datta K. (2016). Relative Biological Effectiveness of Energetic Heavy Ions for Intestinal Tumorigenesis Shows Male Preponderance and Radiation Type and Energy Dependence in APC(1638N/+) Mice. Int. J. Radiat. Oncol. Biol. Phys..

[60] Wong T.P.W., Law Y.L., Tse A.K.W., Fong W.F., Yu K.N. (2010). Influence of Magnolol on the bystander effect induced by alpha-particle irradiation. Appl. Radiat. Isot..

[61] Nagasawa H., Little J.B. (1992). Induction of sister chromatid exchanges by extremely low doses of alpha-particles. Cancer Res..

[62] Ponnaiya B., Suzuki M., Tsuruoka C., Uchihori Y., Wei Y., Hei T.K. (2011). Detection of chromosomal instability in bystander cells after Si490-ion irradiation. Radiat. Res..

[63] Suzuki M., Tsuruoka C. (2004). Heavy charged particles produce a bystander effect via cell-cell junctions. Biol. Sci. Space.

[64] Marín A., Martín M., Liñán O., Alvarenga F., López M., Fernández L., Büchser D., Cerezo L. (2014). Bystander effects and radiotherapy. Rep. Pract. Oncol. Radiother..

[65] Dong C., He M., Ren R., Xie Y., Yuan D., Dang B., Li W., Shao C. (2015). Role of the MAPK pathway in the observed bystander effect in lymphocytes co-cultured with macrophages irradiated with gamma-rays or carbon ions. Life Sci..

[66] Han W., Zhu L., Jiang E., Wang J., Chen S., Bao L., Zhao Y., Xu A., Yu Z., Wu L. (2007). Elevated sodium chloride concentrations enhance the bystander effects induced by low dose alpha-particle irradiation. Mutat. Res..

[67] Kanasugi Y., Hamada N., Wada S., Funayama T., Sakashita T., Kakizaki T., Kobayashi Y., Takakura K. (2007). Role of DNA-PKcs in the bystander effect after low- or high-LET irradiation. Int. J. Radiat. Biol..

[68] Fang Z., Xu A., Wu L., Hei T.K., Hong M. (2016). The role of protein kinase C alpha translocation in radiation-induced bystander effect. Sci. Rep..

[69] Zhou H., Ivanov V.N., Gillespie J., Geard C.R., Amundson S.A., Brenner D.J., Yu Z., Lieberman H.B., Hei T.K. (2005). Mechanism of radiation-induced bystander effect: Role of the cyclooxygenase-2 signaling pathway. Proc. Natl. Acad. Sci. USA.

[70] Zhou H., Ivanov V.N., Lien Y.C., Davidson M., Hei T.K. (2008). Mitochondrial function and nuclear factor-kappaB-mediated signaling in radiation-induced bystander effects. Cancer Res..

[71] Tomita M., Matsumoto H., Funayama T., Yokota Y., Otsuka K., Maeda M., Kobayashi Y. (2015). Nitric oxide-mediated bystander signal transduction induced by heavy-ion microbeam irradiation. Life Sci. Space Res..

[72] Hong M., Xu A., Zhou H., Wu L., Randers-Pehrson G., Santella R.M., Yu Z., Hei T.K. (2010). Mechanism of genotoxicity induced by targeted cytoplasmic irradiation. Br. J. Cancer.

[73] Anzenberg V., Chandiramani S., Coderre J.A. (2008). LET-dependent bystander effects caused by irradiation of human prostate carcinoma cells with X rays or alpha particles. Radiat. Res..

[74] Yin X., Tian W., Wang L., Wang J., Zhang S., Cao J., Yang H. (2015). Radiation quality-dependence of bystander effect in unirradiated fibroblasts is associated with TGF-beta1-Smad2 pathway and miR-21 in irradiated keratinocytes. Sci. Rep..

[75] Schmid T., Multhoff G. (2012). Non-targeted effects of photon and particle irradiation and the interaction with the immune system. Front. Oncol..

[76] Autsavapromporn N., Suzuki M., Funayama T., Usami N., Plante I., Yokota Y., Mutou Y., Ikeda H., Kobayashi K., Kobayashi Y. (2013). Gap junction communication and the propagation of bystander effects induced by microbeam irradiation in human fibroblast cultures: The impact of radiation quality. Radiat. Res..

[77] Shao C., Furusawa Y., Kobayashi Y., Funayama T., Wada S. (2003). Bystander effect induced by counted high-LET particles in confluent human fibroblasts: A mechanistic study. FASEB J..

[78] Kobayashi A., Konishi T. (2018). Radiation quality effects alteration in COX-2 pathway to trigger radiation-induced bystander response in A549 lung carcinoma cells. J. Radiat. Res..

[79] Robbins M.E., Zhao W. (2004). Chronic oxidative stress and radiation-induced late normal tissue injury: A review. Int J Radiat Biol..

[80] Buonanno M., de Toledo S.M., Pain D., Azzam E.I. (2011). Long-term consequences of radiation-induced bystander effects depend on radiation quality and dose and correlate with oxidative stress. Radiat. Res..

[81] Buonanno M., de Toledo S.M., Azzam E.I. (2011). Increased frequency of spontaneous neoplastic transformation in progeny of bystander cells from cultures exposed to densely ionizing radiation. PLoS ONE.

[82] Autsavapromporn N., Plante I., Liu C., Konishi T., Usami N., Funayama T., Azzam E.I., Murakami T., Suzuki M. (2015). Genetic changes in progeny of bystander human fibroblasts after microbeam irradiation with X-rays, protons or carbon ions: The relevance to cancer risk. Int. J. Radiat. Biol..

[83] Wu J., Zhang Q., Wuu Y.-R., Zou S., Hei T.K. (2017). Cytoplasmic Irradiation Induces Metabolic Shift in Human Small Airway Epithelial Cells via Activation of Pim-1 Kinase. Radiat. Res..

[84] Khan M.A., Van Dyk J., Yeung I.W., Hill R.P. (2003). Partial volume rat lung irradiation; assessment of early DNA damage in different lung regions and effect of radical scavengers. Radiother. Oncol..

[85] Calveley V.L., Khan M.A., Yeung I.W.T., Vandyk J., Hill R.P. (2005). Partial volume rat lung irradiation: Temporal fluctuations of in-field and out-of-field DNA damage and inflammatory cytokines following irradiation. Int. J. Radiat. Biol..

[86] Wang T.J., Wu C.C., Chai Y., Lam R.K., Hamada N., Kakinuma S., Uchihori Y., Yu P.K., Hei T.K. (2015). Induction of Non-Targeted Stress Responses in Mammary Tissues by Heavy Ions. PLoS ONE.

